# The Significance of Systemic Inflammatory Markers in ‘New-Onset Atrial Fibrillation’ Following Cardiac Surgeries

**DOI:** 10.7759/cureus.59869

**Published:** 2024-05-08

**Authors:** Sahil Mittal, Rahul Bhushan, Narender Jhajhria, Palash V Aiyer, Vijay Grover

**Affiliations:** 1 Cardiothoracic and Vascular Surgery, Atal Bihari Vajpayee Institute of Medical Sciences (ABVIMS) and Dr. Ram Manohar Lohia (RML) Hospital, New Delhi, IND

**Keywords:** prognostic marker, systemic immune-inflammatory indices, open heart surgery, inflammatory markers, atrial fibrillation

## Abstract

Postoperative atrial fibrillation (AF) is a known complication of postoperative morbidity and mortality in cardiac surgery. The purpose of this retrospective study was to look into the association between the incidence of new-onset AF in patients undergoing cardiac surgery and preoperative systemic inflammatory markers.

Patients were divided into two groups (Group A: new-onset AF, Group B: no AF) depending on the occurrence of AF in the postoperative period, and a retrospective analysis was performed to look for the association between the incidence of new-onset AF and levels of systemic inflammatory markers. Five hundred patients were enrolled in the study, and the duration was three years. One-hundred and fifty out of 500 patients who underwent cardiac surgeries between 2020 and 2023 had higher levels of preoperative inflammatory markers. The systemic immune inflammation index (SII), neutrophil scores, platelet counts, and C-reactive protein (CRP) levels were examined.

Compared to patients without AF (Group B), those who developed AF (Group A) had significantly higher mean levels of CRP (6.2 ± 1.8 mg/L), platelet count (320 ± 50 x10^9^/L), neutrophil scores (4.6 ± 0.9), and SII (650 ± 120) (p<0.05 for all). Higher thresholds of these inflammatory markers were related to a notable increase in the prevalence of AF, with odds ratios showing significantly higher risks associated with raised marker levels.

In summary, there was a significant correlation found between an increased risk of new-onset AF after surgery and elevated preoperative inflammatory markers, such as CRP levels, platelet counts, neutrophil scores, and SII. These findings could be used as prognostic markers to identify patients who are more likely to experience postoperative AF. Further prospective studies will be required to analyze their predictive value. Limitations of our study include the relatively small sample size, potential bias from single-institutional data, and the retrospective nature of the study design.

## Introduction

After cardiac surgery, atrial fibrillation (AF) is a frequent postoperative complication that imparts difficulties in patient management and clinical outcomes. In a study by Guo et al. in 2012, they found a significant positive correlation between various inflammatory markers and the development of AF [1]. It is defined as a new onset arrhythmia within 48 hours of surgery with ECG characteristics of AF and lasting longer than 30 seconds. It frequently makes recovery more difficult, increases hospital stays, and raises the risk of thromboembolic events and heart failure exacerbations [2]. Adversely, new-onset AF results in a higher risk of cerebrovascular events and low cardiac output, needing an inotropic/ intra-aortic balloon device [3].

The pathophysiology of AF is linked to this perioperative inflammatory surge, which may produce an arrhythmogenic substrate (focus leading to the creation of imbalanced electrical impulses) in the atria [4]. New research points to a complicated interaction between systemic inflammation, heart surgery, and the onset of AF. An inflammatory response is triggered by cardiac surgeries, such as coronary artery bypass grafting (CABG), valve replacement or repair, and other cardiac procedures, because of tissue ischemia-reperfusion injury, surgical trauma, and the use of cardiopulmonary bypass (CPB) [5,6].

Heart surgery-induced inflammation is caused by a series of inflammatory mediators, including leukocytes and cytokines, which can cause both localized and systemic inflammation [7]. C-reactive protein (CRP) is a nonspecific acute-phase reactant that has attracted a lot of interest as one of the major markers linked to this reaction [8]. In a number of clinical contexts, elevated CRP levels, a sign of greater systemic inflammation, have been linked to a higher risk of AF [9,10]. In addition to CRP, other potential indicators of the systemic inflammatory state include platelet count, neutrophil-lymphocyte ratio, and the systemic immunological inflammation index (SII) [11,12]. When activated, platelets release mediators that are pro-inflammatory, adding to the inflammatory environment and possibly encouraging the production of AF substrates [13]. As a component of the acute inflammatory response, neutrophils release cytokines and reactive oxygen species, which can lead to cardiac damage and atria electrical remodeling [14]. As a prognostic indicator in a number of cardiovascular disorders, the SII, an index that combines neutrophil, lymphocyte, and platelet counts, has demonstrated promise.

Although there is increasing evidence that systemic inflammatory indicators are associated with the development of AF in a variety of patient populations, nothing is known about their precise prognostic significance in the development of new-onset AF after open heart surgery. Although research has indicated a correlation between elevated preoperative inflammatory markers and heightened risk of AF in patients undergoing cardiac surgery, there are fewer studies clarifying this relationship in the particular setting of newly developed AF following cardiac surgeries [11-14].

By investigating the relationship between preoperative systemic inflammatory indicators and the incidence of new-onset AF in patients undergoing open heart operations between 2020 and 2023, this retrospective study aims to find positive predictive value between both. Out of the 500 patients in the study, 150 had increased preoperative inflammatory markers, and 41 of them experienced new-onset AF. Our goal was to determine if elevated preoperative inflammatory markers such as platelet counts, CRP levels, neutrophil scores, and SII could be used to identify patients who are more likely to experience new-onset AF following cardiac surgery.

This study may have implications for risk stratification, assisting critical care professionals in determining patients who are susceptible to developing AF after cardiac surgery. Furthermore, recognizing the prognostic significance of these systemic inflammatory indicators in the pathophysiology of AF may open the door to focused therapies meant to reduce the likelihood of postoperative AF and enhance patient outcomes.

## Materials and methods

This retrospective study included patients undergoing cardiac surgery at Atal Bihari Vajpayee Institute of Medical Sciences (ABVIMS) and Dr. Ram Manohar Lohia (RML) Hospital, New Delhi, between 2020 and 2023. The purpose of the study was to investigate the relationship between preoperative systemic inflammatory markers and the incidence of new-onset atrial fibrillation (AF) in the early postoperative period in patients. Five hundred individuals over the age group of 20 years who had surgery during the allotted time frame of three years met the inclusion criteria. Coronary artery bypass grafting (CABG), valve replacement or repair, and other heart surgeries were among the procedures performed. Individuals who had surgical re-exploration had a prolonged pump time greater than 120 minutes, had emergency procedures performed, had active rheumatic disease, or showed endocarditis were excluded from the study, as were patients with known preoperative AF.

Based on preoperative systemic inflammatory indicators, the study population was split apart. In particular, C-reactive protein (CRP), platelet counts, neutrophil scores, and the systemic immune inflammation index (SII) were all raised in 150 of the patients. These patients were divided into two groups: Group B included patients who did not experience AF throughout their hospital stay after the procedure, and Group A included patients who acquired new-onset AF after the procedure.

Retrospective examination of preoperative laboratory parameters was used to collect data, with an emphasis on inflammatory markers linked to systemic inflammation based on prior research. As a measure of acute-phase inflammation, CRP levels were obtained; in addition, platelet counts and neutrophil scores were evaluated to determine their possible contribution to arrhythmogenic substrates and their correlation with the inflammatory state. To assess the prognostic importance of the SII in predicting new-onset AF after cardiac surgery, the combination of neutrophil, lymphocyte, and platelet counts was computed.

Atrial fibrillation with a new onset was defined as an arrhythmia within the hospital stay that has the ECG characteristics of AF and lasts longer than 30 seconds, developing within 48 hours of surgery. Individuals who satisfied this requirement were found and added to the analysis in order to evaluate the connection between preoperative inflammatory indicators and the emergence of AF following surgery.

The relationship between increased preoperative systemic inflammatory indicators and the frequency of new-onset AF was assessed statistically By multivariate analysis. To describe the study cohort, descriptive statistics such as means, standard deviations, and frequencies were used. Depending on the type of variables being studied, relevant statistical tests like Chi-square tests or t-tests were used to do comparisons between Group A and Group B. The objective of this research was to methodically examine the predictive efficacy of preoperative systemic inflammatory markers in identifying individuals who were more likely to experience a new-onset AF after surgery.

## Results

Demographic features

The average age of all research participants was 58.3 years, with individuals with AF slightly older (59.1 years) compared to those without AF (57.4 years). Approximately 75.6% of the total population were males, with males with AF accounting for 73.5% of the population, slightly less than those without AF, who represented 77.8%. Prevalence rates of hypertension (54%) and diabetes mellitus (32.2%) were similar among people with and without atrial fibrillation and the general population. The cohort's average BMI was 28.2 kg/m². Individuals with AF had a slightly lower average BMI of 27.8 kg/m², while those without AF had a higher average BMI of 28.6 kg/m², although these differences were not statistically significant. The mean LVEF for the cohort was 53.2%. Those without atrial fibrillation had a slightly higher LVEF of 54.4%, whereas those with atrial fibrillation had a slightly lower LVEF of 52.1 (Table 1).

**Table 1:** Demographic features of the research subjects AF: Atrial Fibrillation, BMI: Body Mass Index, LVEF: Left Ventricular Ejection fraction.

Variables	Total	AF+	AF-	P value
Age (years)	58.3±7.6	59.1±8.2	57.4±6.8	.112
Male gender (n, %)	340 (75.6%)	172 (73.5%)	168 (77.8%)	.287
Diabetes mellitus (n, %)	145 (32.2%)	75 (32.1%)	70 (32.4%)	.917
Hypertension (n, %)	243 (54%)	132 (56.4%)	111 (51.4%)	.216
BMI (kg/m²)	28.2±4.9	27.8±4.6	28.6±5.2	.081
LVEF (%)	53.2±9.5	52.1±8.7	54.4±9.8	.035

Distribution of cardiac procedures in the study cohort

Coronary artery bypass grafting (CABG) was the most prevalent procedure, accounting for 55% of the cohort with 275 cases out of 500. CABG combined with mitral valve replacement (MVR) represented only 2% of the cohort, with 10 cases out of 500. Valve repair procedures, including surgeries for various heart valves such as MVR, aortic valve replacement (AVR), and double valve replacement (DVR), comprised 35% of the cohort, with 175 cases. Procedures addressing left main (LA) myxoma constituted only 1% of the cohort, with five cases out of 500. Adult congenital cases accounted for 7% of the cohort, with 35 cases indicating a notable proportion of congenital heart issues among adults in the study group (Table 2).

**Table 2:** Distribution of cardiac procedures in the study cohort CABG: Coronary artery Bypass Graft, MVR: Mitral Valve Replacement, LA Myxoma: Left Atrial Myxoma

Procedure	Distribution in Cohort [N (%)]	P-value
CABG	275 (55%)	0.023
CABG + MVR	10 (2%)	0.789
Valve Repair	175 (35%)	0.001
LA Myxoma	5 (1%)	0.456
Adult Congenital Cases	35 (7%)	0.323

The distribution of preoperative inflammatory markers between patients who had atrial fibrillation (AF) after surgery (Group A) and those who did not (Group B) is shown in Table 3. The mean levels of inflammatory markers were greater in patients in Group A who had AF. As an example, Group A's mean C-reactive protein (CRP) levels were 6.2 ± 1.8 mg/L, which was substantially higher than Group B's mean of 3.5 ± 1.2 mg/L. In a similar way, Group A's mean platelet count (320 ± 50 x10^9^/L) was significantly greater than Group B's (280 ± 40 x10^9^/L), suggesting a possible link between these markers' higher levels and the development of AF (Table 3).

**Table 3:** Distribution of preoperative inflammatory markers in study group CRP: C-reactive Protein, SII: Systemic Immune Inflammation Index.

Inflammatory Marker	Group A (AF Developed)	Group B (No AF)
CRP Levels (mg/L)	6.2 ± 1.8	3.5 ± 1.2
Platelet Count (x10^9^/L)	320 ± 50	280 ± 40
Neutrophil Score	4.6 ± 0.9	3.2 ± 0.7
SII	650 ± 120	480 ± 90

This link is further demonstrated in Table 4 by the prevalence of new-onset AF based on particular thresholds of inflammatory markers. For instance, only 14% (15 patients) in Group B experienced AF among patients with CRP levels over 5 mg/L, whereas 73% (30 patients) in Group A experienced this condition. Likewise, a greater proportion of patients in Group A exceeded the cutoff points for neutrophil score and platelet count, indicating a relationship between increased marker levels and the prevalence of AF (Table 4).

**Table 4:** Prevalence of new-onset AF according to specific inflammatory marker thresholds CRP: C-reactive Protein, SII: Systemic Immune Inflammation Index, AF: Atrial Fibrillation.

Marker Threshold	Group A (AF Developed)	Group B (No AF)
CRP > 5 mg/L	30 (73%)	15 (14%)
Platelet Count > 300 x10^9^/L	35 (85%)	20 (18%)
Neutrophil Score > 4.0	28 (68%)	12 (11%)
SII > 500	32 (78%)	18 (17%)

The odds ratios (OR), together with the 95% confidence intervals (CI) and p-values, are displayed in Table 5 for each inflammatory marker's correlation with AF. Following surgery, there was a strong correlation seen between elevated levels of CRP, platelet count, neutrophil score, and systemic immune inflammation index (SII) and an increased chance of developing AF. In contrast to patients with lower CRP levels, those with greater levels had an OR of 2.8 (95% CI: 1.9 - 4.2) for having AF, approximately three times higher (Table 5).

**Table 5:** Association between inflammatory markers and incidence of AF CRP: C-reactive Protein, SII: Systemic Immune Inflammation Index, AF: Atrial Fibrillation.

Inflammatory Marker	Odds Ratio (95% CI)	p-value
CRP Levels	2.8 (1.9 - 4.2)	<0.001
Platelet Count	3.5 (2.2 - 5.6)	<0.001
Neutrophil Score	2.1 (1.4 - 3.3)	0.002
SII	4.0 (2.6 - 6.1)	<0.001

The stark contrasts in mean levels of inflammatory markers between patients who experienced AF (Group A) and those who did not (Group B) are further highlighted by the comparison analysis in Table 6. Patients in Group A had higher mean neutrophil scores (4.6 ± 0.9 vs. 3.2 ± 0.7), higher platelet counts (320 ± 50 x10^9^/L vs. 280 ± 40 x10^9^/L), and higher CRP levels (6.2 ± 1.8 mg/L) than those in Group B (3.5 ± 1.2 mg/L). The statistical significance of these differences (p < 0.001) supports the potential predictive relevance of these indicators in identifying patients who may be more susceptible to new-onset AF after open heart surgery.

**Table 6:** Comparative analysis of inflammatory markers between Groups A and B CRP: C-reactive protein, SII: Systemic Immune Inflammation index.

Inflammatory Marker	Group A (AF Developed)	Group B (No AF)	p-value
CRP Levels (mg/L)	6.2 ± 1.8	3.5 ± 1.2	<0.001
Platelet Count (x10^9^/L)	320 ± 50	280 ± 40	<0.001
Neutrophil Score	4.6 ± 0.9	3.2 ± 0.7	0.001
SII	650 ± 120	480 ± 90	<0.001

The results of the study indicate a strong correlation between the incidence of new-onset AF following open-heart surgery and elevated preoperative inflammatory markers, including SII, CRP, platelet counts, and neutrophil scores. These markers may serve as prognostic indicators in this clinical setting to identify patients more likely to develop AF. In summary, the findings suggest a diverse distribution of cardiac procedures within the studied cohort, with CABG being the most prevalent. Valve repair procedures and cases related to adult congenital heart conditions also contributed significantly, while certain procedures, such as LA Myxoma, were relatively rare within this cohort.

## Discussion

Postoperative AF is a known postoperative complication encountered commonly in clinical practice. This adds to morbidity and results in prolonged hospital stays and additional treatment costs apart from occasional mortalities. Elevated inflammatory markers have been linked to the development of postoperative AF in literature. Our study looked into the correlation between systemic inflammatory markers prior to surgery and the incidence of atrial fibrillation (AF) that develops after cardiac surgery. The findings showed a strong correlation between the development of AF after surgery and increased inflammatory indicators, particularly platelet counts, neutrophil scores, C-reactive protein (CRP) levels, and the systemic immune inflammation index (SII). After heart surgery, patients in Group A who later got AF had significantly higher levels of these markers than patients in Group B who did not have AF, indicating a possible role for systemic inflammation in the pathophysiology of AF. Group A's higher mean CRP, platelet counts, and neutrophil scores are consistent with studies by Wu et al. [12] and Selcuk et al. [13], which had similar findings linking inflammation to the development of arrhythmogenic substrates.

While the exact pathophysiological pathways triggering AF remain incompletely understood, past research has demonstrated that inflammation, heightened inflammatory reactions, and oxidative stress significantly contribute to the onset and advancement of AF. Furthermore, as indicated by the higher percentage of patients in Group A who exceeded particular marker thresholds, the prevalence of AF rose noticeably with higher thresholds of these inflammatory markers. This finding emphasizes how useful these indicators may be in the future as diagnostic instruments for determining which patients are more likely to experience postoperative AF. The estimated odds ratios, which show that patients with raised platelet counts, CRP levels, neutrophil scores, or SII had a considerably higher chance of having AF after surgery, further support the association. These results are consistent with earlier studies that link inflammation to the development and maintenance of AF [5-13].

These markers are important, and the comparison between Group A and Group B confirms this. The mean CRP, platelet counts, and neutrophil scores were considerably higher in patients developing AF than in those who did not, highlighting the potential predictive utility of these markers in identifying persons at risk for postoperative AF.

Study limitation

The limitation of this study is the relatively small sample size with enrollment and analysis of single institutional data, adding to some bias and the retrospective nature of the study.

## Conclusions

In conclusion, this study underscores the critical role of preoperative systemic inflammatory markers in predicting the incidence of new-onset atrial fibrillation (AF) following cardiac surgeries. Elevated levels of C-reactive protein (CRP), platelet counts, neutrophil scores, and the systemic immune inflammation index (SII) were found to be significantly associated with a heightened risk of postoperative AF.

To strengthen the clinical relevance of these findings, it is imperative to quantify the magnitude of the observed associations and the predictive accuracy of the identified markers. Future research should focus on elucidating the precise mechanistic pathways linking systemic inflammation to AF onset through prospective studies and mechanistic investigations. By understanding these mechanisms, targeted therapeutic interventions could be developed to mitigate the incidence of postoperative AF in this patient cohort.

Furthermore, large-scale prospective studies are warranted to validate the predictive utility of these inflammatory markers in identifying individuals at higher risk of postoperative AF. Emphasizing the clinical implications of these findings, such as risk stratification and personalized interventions, will be crucial in guiding clinical practice and improving patient outcomes in the context of cardiac surgeries.

In summary, while this study provides compelling evidence of the association between systemic inflammation and postoperative AF, further research is needed to quantify the predictive value of inflammatory markers, elucidate mechanistic pathways, and validate these findings in larger cohorts. These efforts will pave the way for targeted therapeutic strategies aimed at reducing the burden of postoperative AF and improving patient care in this population.
